# Achieving Population-Level Immunity to Rabies in Free-Roaming Dogs in Africa and Asia

**DOI:** 10.1371/journal.pntd.0003160

**Published:** 2014-11-13

**Authors:** Michelle K. Morters, Trevelyan J. McKinley, Daniel L. Horton, Sarah Cleaveland, Johan P. Schoeman, Olivier Restif, Helen R. Whay, Amelia Goddard, Anthony R. Fooks, I. Made Damriyasa, James L. N. Wood

**Affiliations:** 1 Disease Dynamics Unit, Department of Veterinary Medicine, University of Cambridge, Cambridge, United Kingdom; 2 Animal Health and Veterinary Laboratories Agency, Weybridge, United Kingdom; 3 School of Veterinary Medicine, Faculty of Health and Medical Sciences, University of Surrey, Guildford, United Kingdom; 4 Institute of Biodiversity, Animal Health and Comparative Medicine, University of Glasgow, Glasgow, United Kingdom; 5 Department of Companion Animal Clinical Studies, Faculty of Veterinary Science, University of Pretoria, Pretoria, South Africa; 6 Faculty of Medical and Veterinary Sciences, University of Bristol, Bristol, United Kingdom; 7 Department of Clinical Infection, Microbiology and Immunology, University of Liverpool, Liverpool, United Kingdom; 8 Fakultas Kedokteran Hewan, Universitas Udayana, Bali, Indonesia; University of Edinburgh, United Kingdom

## Abstract

Canine rabies can be effectively controlled by vaccination with readily available, high-quality vaccines. These vaccines should provide protection from challenge in healthy dogs, for the claimed period, for duration of immunity, which is often two or three years. It has been suggested that, in free-roaming dog populations where rabies is endemic, vaccine-induced protection may be compromised by immuno-suppression through malnutrition, infection and other stressors. This may reduce the proportion of dogs that seroconvert to the vaccine during vaccination campaigns and the duration of immunity of those dogs that seroconvert. Vaccination coverage may also be limited through insufficient vaccine delivery during vaccination campaigns and the loss of vaccinated individuals from populations through demographic processes. This is the first longitudinal study to evaluate temporal variations in rabies vaccine-induced serological responses, and factors associated with these variations, at the individual level in previously unvaccinated free-roaming dog populations. Individual-level serological and health-based data were collected from three cohorts of dogs in regions where rabies is endemic, one in South Africa and two in Indonesia. We found that the vast majority of dogs seroconverted to the vaccine; however, there was considerable variation in titres, partly attributable to illness and lactation at the time of vaccination. Furthermore, >70% of the dogs were vaccinated through community engagement and door-to-door vaccine delivery, even in Indonesia where the majority of the dogs needed to be caught by net on successive occasions for repeat blood sampling and vaccination. This demonstrates the feasibility of achieving population-level immunity in free-roaming dog populations in rabies-endemic regions. However, attrition of immune individuals through demographic processes and waning immunity necessitates repeat vaccination of populations within at least two years to ensure communities are protected from rabies. These findings support annual mass vaccination campaigns as the most effective means to control canine rabies.

## Author Summary

Canine-mediated rabies is a horrific disease that claims tens of thousands of human lives every year, particularly in Asia and Africa. The disease can be effectively controlled through mass vaccination of dogs with high-quality vaccines; however, questions remain over the effectiveness of vaccination where the health status of free-roaming dogs may be compromised and the life expectancy and access to these dogs may be limited. This study evaluated rabies-vaccine induced immune responses and vaccine delivery in previously unvaccinated, free-roaming dog populations in two rabies endemic regions in Asia and Africa, to better understand the effectiveness of vaccination campaigns. We found that the majority of dogs seroconverted to the vaccine regardless of health status. Excellent vaccination coverage was achieved through community engagement and door-to-door vaccine delivery, even where the majority of the dogs needed to be caught by net for vaccination. However, attrition of immune individuals through demographic processes and waning immunity reinforces the importance of frequent and regular vaccination campaigns to ensure effective vaccination coverage is maintained.

## Introduction

Canine-mediated rabies is a viral zoonosis, causing at least 55,000 human deaths every year [1]. Mortality from rabies is highest in less developed communities in Asia and Africa, where domestic dogs are free-roaming [2]–[8]; with increasing evidence that the majority are owned [2], [3], [6], [9], [10] and, thus, generally accessible for vaccination [11], [12].

Canine rabies can be effectively controlled by vaccination [13]–[16] using readily available, high potency (antigenic value ≥1 IU/ml), inactivated cell-culture vaccines. These vaccines should provide protection from challenge in healthy dogs for the claimed period for duration of immunity [17], which is often two or three years. In free-roaming dog populations, vaccine-induced protection from rabies may be compromised for several reasons. These include: (a) insufficient vaccine delivery during vaccination campaigns [11], (b) lack of repeat vaccination campaigns, with loss of vaccinated individuals from populations through demographic processes [18], [19], and a substantial proportion of dogs probably vaccinated only once in their lifetime [20], despite them often living beyond three years of age [19]; and, (c) the possibility of immuno-suppression through malnutrition, infection or other stressors [21]–[23], which may reduce the proportion of dogs that seroconvert or the duration of immunity of those dogs that seroconvert. These constraints may result in a decline in the vaccination coverage between campaigns to below 20–45%, the threshold necessary to control rabies [24]. Consequently, investigating the effectiveness of vaccination campaigns under field conditions is critical.

The adaptive (B-cell humoral and T-cell cell-mediated) immune response to vaccination is complex. The humoral response generates virus neutralizing antibody (VNA), the primary correlate of protection induced by viral vaccines [18], [25]–[27]. Cell mediated immunity (CMI) is also important for the development of vaccine-induced immunity [28]–[30] and acts in synergy with the humoral response [27]. Ongoing protection from challenge depends on the persistence of long-lived plasma cells, continuing to generate antigen-specific antibody, and B- and T- memory cells. The primary antibody response following vaccination generally correlates with the strength of the memory response (B- and T-cell) and, thus, the ability to induce secondary responses to subsequent challenge [27], [31]–[35]. In healthy dogs the quality of the primary immune response to vaccination depends on several factors, including the type of vaccine, with modified-live vaccines generally inducing superior responses, the route of administration, and the dose of vaccine antigen [25], [27], [32], [33], [35]–[37].

Laboratory challenge studies in healthy dogs support these observations. Following seroconversion, protection from rabies virus challenge correlates with peak VNA titre and final titre prior to challenge for inactivated, DNA and modified-live vaccines, with increased susceptibility to challenge once titres drop to near negligible levels (VNA titres <0.1 IU/ml or mouse serum neutralizing antibody titres <1∶2 dilution) [31]–[35], [37]–[41]. These studies used comparable antibody assays [42], [43] and virus challenge doses. Titres measured repeatedly over 3–4 years initially peaked and then declined rapidly, followed by a more gradual decline [31], [33], [34], [44]. While a titre of 0.5 IU/ml demonstrates seroconversion following vaccination [45], the approximate threshold for protection following seroconversion may be 0.1 IU/ml [34], [37], [40], [46]. However, in the aforementioned experimental studies, only a proportion (<40%) of dogs with measureable titres following vaccination, but with negligible titres at the time of challenge succumbed to challenge, highlighting the importance of previously activated B- or T- cells allowing rapid response to challenge.

Although the same relationship between VNA titre and protection from challenge is expected in immuno-suppressed dogs as in healthy dogs [21], [23], no systematic comparison has been published to date. Reduced humoral immune responses have been shown in malnourished experimental dogs [22] and Gambian children vaccinated with human diploid-cell rabies vaccine [47], and pet dogs with anaemia or intestinal parasites vaccinated against rabies [37], [48]. Several studies have evaluated the immune response in previously unvaccinated, mostly healthy pet dogs to high potency, inactivated rabies vaccine under field conditions [48]–[54]. All of these studies report variable VNA titres up to 12 months following vaccination, including a proportion of dogs with titres ≤0.1 IU/ml (and generally a larger [17% to >42%] proportion with titres <0.5 IU/ml). These observations have serious implications for free-roaming dogs where their health status is more likely to be compromised. However, with the exception of one study in Peru [55], no study has evaluated variations in vaccine-induced VNA in previously unvaccinated free-roaming dogs where rabies is endemic. Furthermore, no study has properly evaluated the factors associated with these variations.

Cell mediated immunity is technically difficult to measure under field conditions [28], [56], however peripheral blood lymphocyte counts, which are predominately T-cells [57], may provide a straightforward, indirect assessment of CMI. Together with cytokine assays and measures of blastogenic responses of lymphocytes to mitogen, lymphocyte counts were used to assess immunomodulation in healthy dogs in response to vaccination [58]–[60] and protein-calorie malnutrition [22], and in humans in response to protein-calorie malnutrition [61]. In dogs, malnutrition induced declines in immunoglobulin and lymphocyte function and counts. Therefore, lymphocyte counts together with rabies vaccine-induced titres and nutritional status may correspond to the overall immune status of an individual and susceptibility to infection.

This study focused on evaluating temporal variations in vaccine-induced VNA, and factors associated with these variations, in three previously unvaccinated, owned free-roaming dog populations in South Africa and Indonesia, to better understand their effect on vaccination coverage. In addition, the efficiency of vaccine delivery and loss of vaccinated individuals from the cohorts were also assessed.

## Materials and Methods

### Study populations

See Table S1 for a summary of the methodology. Data were collected from three cohorts of dogs, one in South Africa, and two in Indonesia. The cohorts were part of a larger ecological study that commenced in March 2008 [19]. The South African cohort was located in Zenzele, an informal settlement 10 km west of Johannesburg (26.15°S and 27.41°E). In Indonesia the cohorts were located in the study areas of Kelusa (8.26°S and 115.15°E) and Antiga (8.30°S and 115.29°E), two villages on the island of Bali. Kelusa, composed of six banjars (sub-villages), is inland. The study area encompassed the entire village except for Banjar Yehtengeh, separated from the rest of the village by rice fields and jungle, the southern half of Banjar Kelikikawan and the households scattered along the main road leading into the village. Antiga, a large village of six banjars, is located on the east coast. The bulk of the households are clustered into two banjars (Kaler and Kelod). The study area encompassed all of Kaler and Kelod. An additional area (Banjar Ketug) included households scattered along a 2.7 km stretch of road winding through the jungle north of Kaler and Kelod. Rabies is endemic in Indonesia and South Africa, with outbreaks occurring in Bali in 2008 and Gauteng Province in 2010.

The Zenzele research cohort included every available dog in the entire township (which was the study area) in February 2010 that had not been previously vaccinated by the Department of Agriculture (DoA) during a vaccination point (VP) on the outskirts of the township in October 2009 (Table S2). All the dogs vaccinated by the DoA were identified within one week of the one day VP through a rapid door-to-door search, with verification by owners and inspection of certificates. The DoA had also set-up a VP on the outskirts of Zenzele in May 2006, thus vaccination history and certification were checked with each owner at the start of the study. VNA titres were also evaluated for anamnestic responses to vaccination consistent with previous vaccination.

The Bali research cohorts included every available dog in the study areas of Kelusa and Antiga in January 2010 that had not been previously vaccinated by the Department of Livestock (DoL) as described below (Table S2). Prior to a rabies outbreak in 2008, vaccination against rabies was illegal in Bali and there had been no systematic vaccination programs in either village prior to commencement of the study. Vaccination points were set up by the DoL in two banjars in Kelusa in December 2009 and in one banjar outside of the study area in Antiga in February 2010. The VPs were poorly attended because of community awareness of the research vaccination program and because the owners could not readily handle their dogs. In Kelusa, 16 dogs from the study area attended the vaccination points. In Antiga only three dogs from the study area attended the vaccination point.

All of the dogs resident in the study area were owned and had been previously identified by household, name and appearance through intensive monitoring by direct observation and survey since March 2008. Intensive monitoring of all of the dogs in the study area continued until April 2011. Therefore, all of the dogs in the study population were readily identified at the individual level during the study period. There was no evidence for a resident population of unowned dogs [19], [62]. All dogs in their third month of life or older were photographed (standardised dorsal and lateral views). Pups in their first or second month of life were recorded but not photographed. The same enumerators had tracked the majority of the cohorts at the individual level since March 2008 and were familiar with the dogs.

### Vaccination and sampling

Vaccine delivery was door-to-door for the research cohorts, and households were revisited repeatedly until the dog was caught for vaccination and blood sampling, or it was apparent that the dog could not be caught or the owner would not be available to give consent. A dog was also excluded from the study if the owner declined consent, the dog did not remain calm during restraint, there was a high index of suspicion that the dog may bite, or it was apparent the dog had a clinical condition that might have deteriorated as a result of restraint.

All the dogs were carefully restrained by experienced personnel using the correct equipment and under the direct supervision of a veterinarian. In Zenzele, dogs were gently restrained with a leash and soft muzzle. In Bali most dogs could not be safely restrained by leash and muzzle and required restraint by net. Vaccinations and blood sampling were undertaken by experienced veterinarians. High-quality, sterile consumables (i.e. needle, syringe and blood tubes) were used for each vaccination and blood sample. Dogs in the research cohorts were vaccinated with 1 ml of Rabisin [63], an inactivated rabies vaccine containing at least 1 IU/ml of rabies virus glycoprotein (GS57 Wistar strain) with an aluminium hydroxide adjuvant. Vaccine was administered subcutaneously into the neck or shoulder region. The vaccine cold chain was carefully preserved.

Rabisin and Galaxy DA2PPv, a polyvalent vaccine against common infectious pathogens, was administered by the DoA during the October 2009 VP in Zenzele. Some dogs vaccinated at the VP may have received ivermectin. The DoL administered Rabisin during the February 2010 VP in Antiga, and Rabivet Supra 92, a locally produced cell-culture vaccine, during the December 2009 VP in Kelusa. Vaccine administration and storage by the local authorities were not observed.

Different blood sampling schedules were required for Zenzele and Bali given the different methods of restraint and because the rabies outbreak in Bali escalated during 2009, forcing vaccination to be undertaken 6 months earlier than planned. Every dog in each research cohort, including neonates, was vaccinated at the start of the study (day 0) (Zenzele n = 259, Kelusa n = 284 and Antiga n = 259 vaccinated [Table S2]), and every available dog from about 6–8 weeks of age was blood sampled (see Table 1 and Table S3 for the number of dogs blood sampled at each time point).

**Table 1:** The number of dogs in the research cohorts and the number of unvaccinated controls in Bali that were blood sampled at each time point (this table is reproduced with additional information in the Supporting Information Table S3).

	day 0*	day 30	day 90	day 180	day 360
Zenzele vaccinated dogs	190	183	148	134	103
Kelusa vaccinated dogs	_	_	_	168	124
Kelusa unvaccinated dogs	_	_	_	70	79
Antiga vaccinated dogs	_	_	_	163	126
Antiga unvaccinated dogs	_	_	_	35	49

*day 0 immediately prior to vaccination for the research cohort.

Blood was collected from the Zenzele research cohort on day 0 (immediately prior to vaccination) and then approximately 30, 90, 180 and 360 days following vaccination. The dogs vaccinated by the DoA were also blood sampled 8–10 days after the VP. Samples were then collected approximately 30, 90, 180 and 360 days following the VP. In Zenzele, only those dogs that had been vaccinated were blood sampled. Rabies-vaccine induced VNA was measured at each time point. Complete blood counts (CBCs) were measured on days 0, 180 and 360 for the research cohort.

In Bali, samples were collected on day approximately 180 and 360 following vaccination. Every available dog, whether vaccinated or not, was blood sampled at both time points and analysed for rabies-vaccine induced VNA. Unvaccinated dogs constituted the control group, and included those dogs not caught for vaccination on day 0 and those that arrived into the study populations after day 0. The sixteen dogs in Kelusa and three dogs in Antiga vaccinated by the DoL, in December 2009 and February 2010 respectively, were blood sampled at the same time as the research cohort.

In all the sites, households were visited in approximately the same order at each time point, so the number of days between samples were similar for each dog.

For each sample, 5–7 ml of blood was collected from the jugular or cephalic vein and divided into plain and ethylene diamine-tetraacetic acid (EDTA) containing blood tubes. The blood tubes were immediately coded by date, house number and dog identification and placed in cool boxes with ice packs. Serum was separated by centrifugation within 8 hours of collection and refrigerated at 4–6°C for up to 48 hours prior to freezing. All the sera were transported frozen in dry shippers to the Weybridge Animal Health Veterinary Laboratory Agency in the United Kingdom for fluorescent antibody virus neutralization (FAVN) assays. EDTA whole blood samples were refrigerated and then tested within 48 hours of collection for CBCs. Approximately 10 grams of faeces was collected manually on day 0 from 107 dogs randomly selected from the Zenzele cohort for routine analysis. Upon collection, the faecal sample pots were similarly coded and kept in the cool boxes, then refrigerated until being tested. Complete blood counts and faecal analysis were undertaken by the Faculty of Veterinary Science, University of Pretoria. Suitable laboratory facilities were not accessible in Bali for these tests. Finally, 32 dogs from Kelusa and Antiga combined were selected on day 180 from those dogs diagnosed with generalised dermatitis during the preceding survey for deep skin scrapes (DSS) from affected areas of skin to determine the prevalence of *Demodex spp*. See text S1 and text S3 for an explanation of sample selection for the DSS and faecal analysis.

### Covariates

Factors that may influence the immune response to rabies vaccine were selected on their measurability under field conditions, particularly by vaccinators. These factors had been previously quantified at the individual level as part of the larger ecological study that commenced in March 2008, and the methods used to quantify the factors are described elsewhere [19]. In summary, the factors were categorical and measured by direct observation and questionnaire at the time of vaccination (gender, age class, pregnancy, lactation, sterilisation status [Bali only], intestinal parasites [Zenzele only]) or within 6 weeks of vaccination (body condition, clinical signs associated with serious illness, protein intake [Bali only], and generalised dermatitis [Bali only]) [21], [22], [37], [47], [48], [64]–[67]. See text S2 and Table S19 for a detailed description of the covariates. Time (points) was treated as a continuous variable.

### Analytical methods

#### Laboratory tests

VNA was measured by fluorescent antibody virus neutralization (FAVN), a method prescribed by the Office International des Epizootes (OIE) [42]. In order to evaluate the variability in titres, including ≤0.1 IU/ml, the assay was modified to include a two-fold dilution with reciprocal dilutions ranging from 2 to 4096. Fifty percent endpoint titres, estimated by the Spearman-Karber method [68], were converted into international units (IU/ml) by comparison with a standard serum. All samples were tested within two weeks of thawing and re-frozen within three weeks of testing. Except during assay preparation, all thawed samples were refrigerated.

All of the samples from the same dog were tested within the same batch. Consequently, samples from each dog were frozen for a variable amount of time between collection and testing and a proportion of the samples were stored for over 12 months. To evaluate the effect of storage time and freeze-thaw cycles on titres, 25 samples were randomly selected from the first batch tested. These samples had been frozen (−20°C) for over 2 years between the initial and repeat tests.

To rule out cross-reaction with Lyssaviruses other than Rabies Virus (RABV), 30 samples were randomly selected from the Zenzele research cohort (day 0) and 60 from the Bali research cohorts and controls (day 180 and 360) combined and tested against Lagos Bat Virus (LBV), an antigenically divergent virus from Phylogroup II Lyssaviruses [69].

Complete blood counts were determined by an automated cell counter (ADVIA 2120 Siemens) using impedence counting, flow cytochemistry, laser light scattering and validated veterinary package software. The differential leukocyte counts were confirmed by manual counting.

Deep skin scrapes and faecal samples were evaluated using standard protocols [65], [70] (text S1).

#### Statistical methods

A range of models were used to explore the relationship between time after vaccination and physiological and health status at the time of vaccination on titre. Correlation coefficients for titres and log titres were determined for a combination of time points (i.e. day 30, 90, 180 and 360) for the vaccinated dogs in Zenzele. This suggested that dogs with higher peak VNA titres also had higher titres towards the end of the study period.

Linear mixed effects models were fitted to the longitudinal data from the vaccinated dogs in the research cohorts using the nlme package in R (3.0.1) [71], [72]. Dogs vaccinated by the local authorities in Zenzele in October 2009, Kelusa in December 2009 and Antiga February 2010 were excluded from these analyses because the administration of a standardised dose of Rabisin was not observed. The response variable, of VNA titre (here after referred to as “titre”) following vaccination, was modelled as the natural log of the titre (determined by Box-Cox transformation) expressed in IU. Therefore, baseline (day 0) titres were dropped from the Zenzele models and the unvaccinated (control) dogs were excluded from the Bali models. Explanatory variables included time (points) and the covariates described under *Covariates* (also see text S2 and Table S19) as fixed effects, and dog as a random effect. All individuals with complete information for the variables of interest were included in the models. Forward and backwards stepwise regression compared the full range of covariates and their biologically plausible interactions to the null model. The models with the lowest Akaike's Information Criteria (AIC) for the highest number of observations were retained.

Models were first fitted to each cohort separately. The Bali cohorts were then combined and the model refitted with dog nested within study area (i.e. village) as a random effect. Finally, all the research cohorts were combined and the models refitted. Each of these models were fitted with and without upper outliers (i.e. day 30 titres ≥128 IU/ml for 7 dogs in Zenzele, and day 180 titres ≥11.3 IU/ml for 4 dogs in Kelusa and 15 dogs in Antiga) in order to exclude dogs from the analysis that may have been previously vaccinated by the DoA in Zenzele in May 2006, as part of vaccination campaigns outside of Kelusa and Antiga, or privately by their owners. Upper outliers were defined according to vaccination history, breed, age, source, geographical location and post-vaccinal titres (further described under *Assessment of prior vaccinations* in the Results).

**Figure 1:**
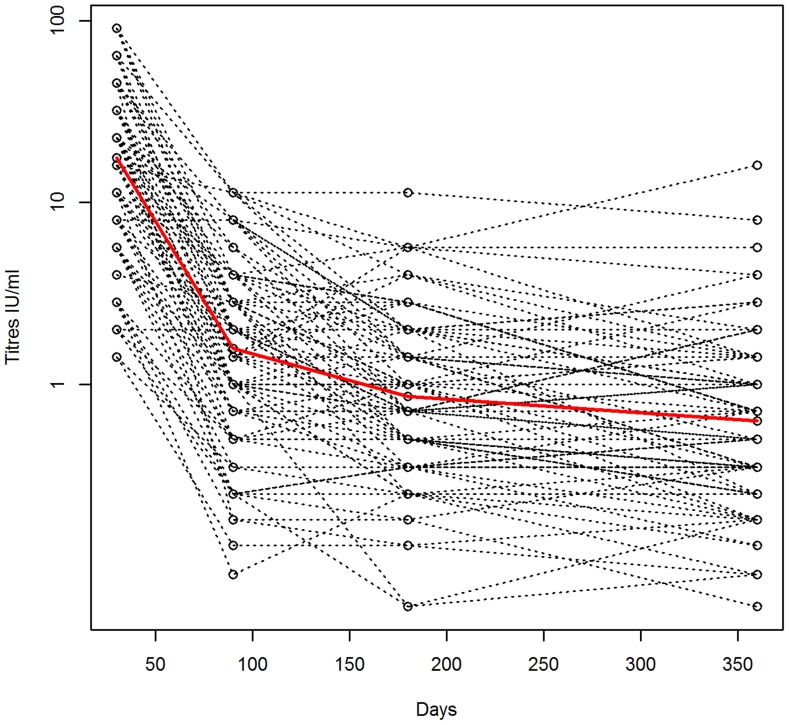
Declines in titre in the Zenzele research cohort. Titres of all the dogs (n = 82) in the Zenzele research cohort that were blood sampled all at four time points (30, 90, 180 and 360 days after vaccination). Upper outliers (i.e. the dogs with day 30 titres ≥128 IU/ml) are excluded. Titres in IU/ml are shown on the log scale. The geometric mean titre is shown in red.

The models take the form:
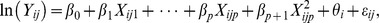
where 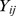 is titre and 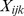 (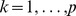) are the covariates for observation 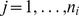 on individual 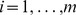, where the final covariate (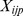) is time. Hence, time is modelled as a quadratic curve (Figure 1). The vector 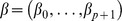 is a vector of regression coefficients, and the vector 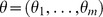 corresponds to a set of individual-level random effect terms, such that 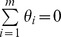. Finally the error terms 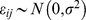.

This model was fitted to the full data set for Zenzele, which included all the time points (i.e. day 30, 90, 180 and 360). The data set included one to four data points for each individual depending on the availability of the individual for blood sampling during the study period. Times were adjusted by 30 days to allow the model intercepts to correspond to day 30 (peak) titres. A model using an exponential decay (rather than quadratic) over time was also fitted, however the quadratic model provided a marginally better fit to the data across this range, and so only the results from the quadratic model are reported here.

The Bali data contained only one or two data points for each individual (i.e. day 180 and 360), and so instead a linear relationship to time was used (instead of quadratic). To facilitate comparisons with the Bali cohorts, linear models were fitted to truncated data sets for Zenzele (i.e. day 90 or 180 to 360), and the time (points) were adjusted to allow the intercepts to correspond to titres on day 90 or 180 respectively. Experimental studies report a spike in titre immediately following vaccination, followed by a prolonged, slow decline in titre [34]. Although a quadratic relationship with time fits the Zenzele data set well over the observed range of the data (Table S18), it does not monotonically decrease over time and hence is a poor choice for predictions beyond the range of the data. Exponential decay models do decease monotonically, but do not have heavy enough tails given how we would expect the titres to decay outside the range of the data, based on previous studies [34]. Fitting alternative models to a skewed distribution with heavy tail for predictions is challenging given that there is insufficient data in the extremes in order to robustly estimate the tail. Therefore, linear models, fitted to Zenzele data sets that exclude peak (day 30) titres, were selected to approximate prolonged, slow declines in titre for predictions in GMT beyond the last time point (Table S17).

To explore the relationship between the natural log of the day 30 (peak) titres and the covariates described under *Covariates* and lymphocyte and eosinophil counts on day 0, 180 and 360 for Zenzele, linear models were fitted to these data and model selection performed using stepAIC with the MASS package in R (3.0.1) [73]. These models were equivalent to an analysis of variance. An association between eosinophil counts and antibody titres at each time point was also assessed.

The final models were checked for violation of constant variance and normal error distribution assumptions.

Mann-Whitney tests were used to compare titres between (a) vaccinated dogs in Zenzele, Kelusa and Antiga for the same time points, (b) unvaccinated dogs in Kelusa and Antiga for the same time points, and (c) dogs present in Zenzele in May 2006 and those that arrived into the population after May 2006. The natural log of the titre was used for these comparisons. The Mann-Whitney test was also used to compare peak (day 30) titres between dogs with day 360 titres <0.5 IU/ml and ≥0.5 IU/ml in Zenzele, and the cube root transformation of the titre was used to stabilise the variance according to a Box-Cox transformation. Population structures were stable [19], therefore age-specific life expectancies were estimated, by standard analysis of vertical life tables [9], [74], [75], from the observed ages of the entire study population at the end of the study period.

### Ethics

The study was approved by the Ethics Committee, University of Cambridge [DVM/EC/1-2010], and the Animal Ethics Committee, University of Pretoria [v025-10 AUCC]. Permits to collect demographic data were granted by the Ministry for Research and Technology (RISTEK), Indonesia [03923/SIP/FRP/SM/IV/2010]. Blood samples were collected under the auspices of the Faculty of Veterinary Medicine, Udayana University, Bali [RG49780], and permits for vaccination and blood collection were granted by the Balinese provincial and regencies Departments of Livestock, the districts Centres of Animal Health (UPT) [RG49780], and Kesbang, Pol and Linmas (the combined Agencies for National Unity, Politics and Protection) [070/607.D.III and 070/015/D.II]. In all of the sites, informed consent was obtained prior to each survey and blood test from the community leaders and owners, who were kept fully informed of the purpose, approach and progress of the study. Vaccination and blood sampling were only carried out with the owner, or responsible adult delegated by the owner, present and their express consent.

## Results

### General description of the study populations

Almost all of the dogs in the study populations were owned but free-roaming, with <10% confined continuously or frequently during the study period March 2008–April 2011. There was an approximately even ratio of male to female dogs in Zenzele, but the ratio was skewed towards males (approximately 75%) in Bali. Less than 2% of dogs were sterilised in Zenzele, but castration of juvenile male dogs by community members was common in Bali (approximately 14% in Kelusa and 27% in Antiga) [19]. Life expectancy was at least 3 years for the majority of dogs in the study populations (Table S4 and Figures S1a–S1c).

### Vaccination coverage

High vaccination coverage was achieved through door-to-door vaccine delivery: 82% (259/315) in Zenzele, 81% (284/351) in Kelusa and 79% (259/327) in Antiga. Similar coverage (75–86%) was achieved in Bali for blood sampling at day 180 and 360, despite many of the dogs having been caught on at least one previous occasion (Table S2). The characteristics of dogs that avoided capture are described in Table S5. The sex ratio and age distribution of these dogs were similar to the overall population (Figures S1a–S1c).

Attrition of the cohorts occurred during the study period through mortality, particularly of neonates, but also through the relocation and disappearance of dogs [19]. Of the 259 dogs vaccinated in Zenzele at the start of the study, 103 (40%) were sampled at the last time point. Similar proportions were recorded in Kelusa (44%, n = 124) and Antiga (49%, n = 126) (Tables S2 and S3).

### Assessment of prior vaccinations

In the Zenzele research cohort, upper outliers were defined as dogs with peak titres (on day 30) of 128 IU/ml or greater (n = 7). Some of these dogs were either in the study area in May 2006 or may have been previously independently vaccinated by their owner. Baseline titres of the upper outliers were ≤0.25 IU/ml, most with a titre of ≤0.09 IU/ml. The history of those individuals with the next highest titre (91 IU/ml) varied, and included seven dogs that were born in Zenzele after October 2009.

It is unlikely that any of the dogs vaccinated by the DoA four months prior to initiation of vaccination of the research cohort were inadvertently included in the research cohort. The day 0 titres of the research cohort (including upper outliers ranged from 0.06–1 IU/ml with a GMT of 0.1 IU/ml) were substantially lower than the day 90 titres of the DoA cohort (including upper outliers ranged from 0.06–128 IU/ml with a GMT of 2.8 IU/ml). Thirteen (20%) of the dogs vaccinated by the DoA had titres ≤1 IU/ml 90 days after vaccination, of which 6 had titres <0.5 IU/ml and four of these were non-responders (i.e. day 30 titre of <0.5 IU/ml). Only five dogs in the research cohort had day 0 titres ≥0.5 IU/ml, and of these none appeared to have an anamnestic response to the vaccine (day 30 titres ranged from 1.4–45 IU/ml) (Tables S6 and S11). There were no differences in the distributions of titres for dogs in Zenzele probably present in May 2006, when the DoA vaccinated, and those that arrived into the population after May 2006 (Table S12).

In the Bali research cohorts, upper outliers were defined as dogs with day 180 titres of 11.3 IU/ml or greater (n = 4 in Kelusa; n = 15 in Antiga). For some of these dogs, information provided by their owner, breed, source and geographical location was suggestive of vaccination undertaken independently by their owner or as part of vaccination campaigns outside of Kelusa and Antiga. Several (n = 15) unvaccinated controls had titres ≥0.5 IU/ml (Tables S7, S8, S9). The titres of the unvaccinated controls are summarised in Table S10.

### Evaluation of antibody titres

#### Titre variations in the vaccinated dogs

**Figure 2:**
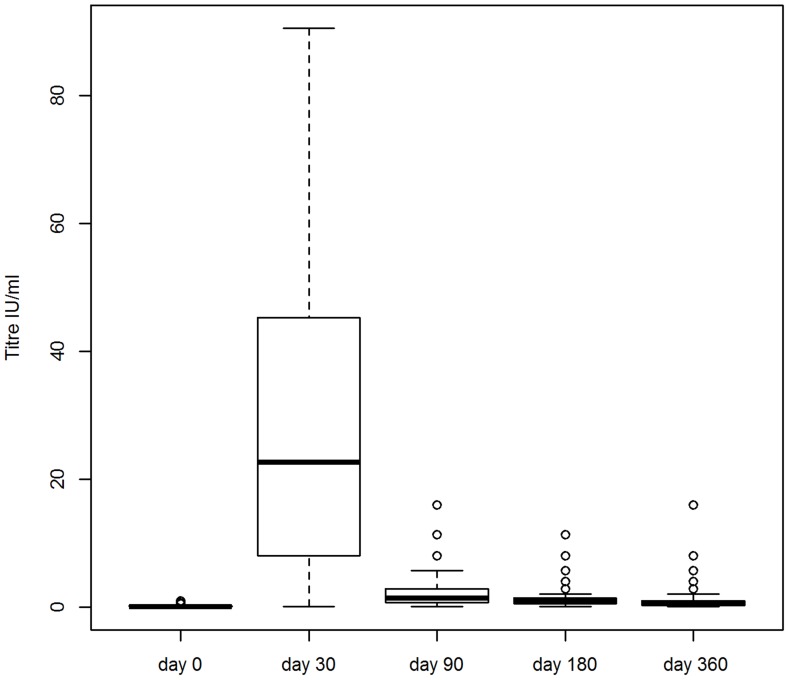
Variations in titre in the Zenzele research cohort. Titres of all the dogs in the Zenzele research cohort. Upper outliers (i.e. the seven dogs with day 30 titres ≥128 IU/ml) are excluded. The median titre (thick, horizontal line), 25th and 75th percentiles (thin horizontal lines), and either minimum and maximum titres or 1.5× the interquartile range (dashed vertical lines) are shown for each time point after vaccination (at day 30, 90, 180 and 360). Day 0 shows the distribution of titres immediately prior to vaccination.

**Figure 3:**
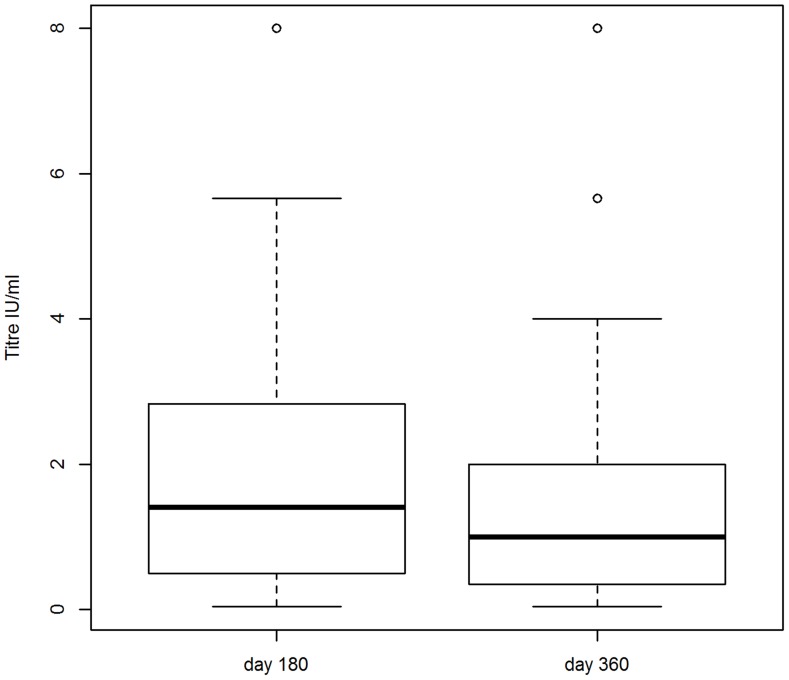
Variations in titre in the Kelusa research cohort. Titres of all the dogs in the Kelusa research cohort. Upper outliers (i.e. the four dogs with day 180 titres of 11.3 IU/ml) are excluded. The median titre (thick, horizontal line), 25th and 75th percentiles (thin horizontal lines), and either minimum and maximum titres or 1.5× the interquartile range (dashed vertical lines) are shown for each time point after vaccination (at day 180 and 360).

**Figure 4:**
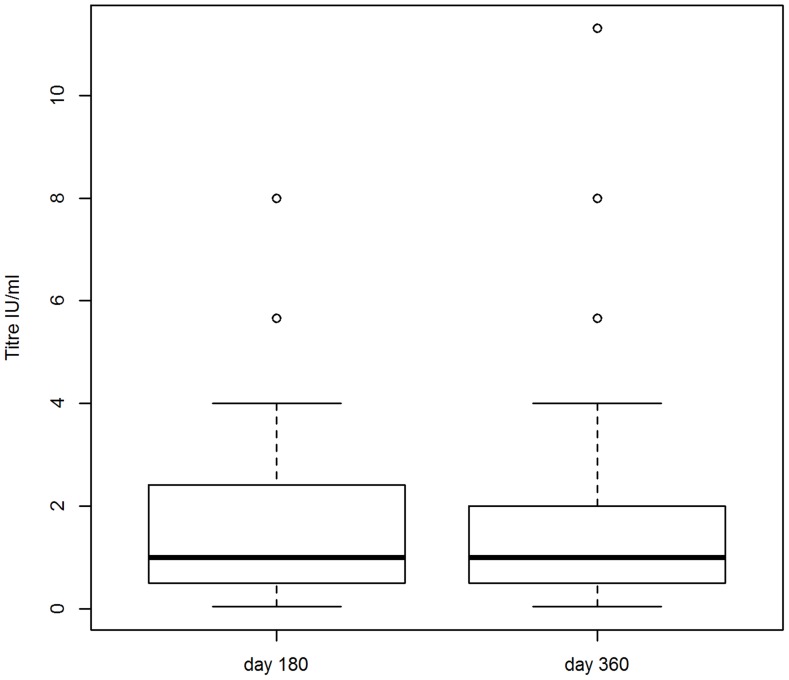
Variations in titre in the Antiga research cohort. Titres of all the dogs in the Antiga research cohort. Upper outliers (i.e. the fifteen dogs with day 180 titres of 11.3 IU/ml) are excluded. The median titre (thick, horizontal line), 25th and 75th percentiles (thin horizontal lines), and either minimum and maximum titres or 1.5× the interquartile range (dashed vertical lines) are shown for each time point after vaccination (at day 180 and 360).

The quality of the serum samples was excellent, with only a few samples with slight to moderate haemolysis. Most dogs in Zenzele seroconverted (97% of the research and 92% of the DoA cohorts had titres ≥0.5 IU/ml at day 30), however there was considerable variability in titres at each time point (Figure 2). The estimated dog-dog variation (random effect) in peak titres (quadratic model intercept) was large (+/− 2SD 1.8–99 IU/ml) (Table S17, model 1). Excluding upper outliers, the observed geometric mean titres (GMT) at day 30 for the research cohort (of 15 IU/ml, Table S18) was comparable to experimental [34], [35] and field [48] studies of previously unvaccinated dogs. The maximum peak titre was more than double the upper limit of the other studies (40–50 IU/ml), however those dogs with peak titres >40 IU/ml included seven dogs born in Zenzele after October 2009 which were unlikely to have been vaccinated prior to commencement of the study. There was similar variability in the titres at each time point for the Bali cohorts (Figures 3–4; Table S17, models 3–6). See Table S13 for details of the dogs in Zenzele that did not seroconvert to the vaccine.

Although the GMTs for the Bali cohorts were statistically significantly higher than Zenzele (Mann-Whitney test p≤0.05) for day 180 and 360, the value of the means and modes were comparable between cohorts at each time point (Table S14). Less than 10% of each cohort had titres of ≤0.1 IU/ml at day 360. For Zenzele, peak titres did not exceed 5.7 IU/ml for these dogs, and three were non-responders. See Table S15 for details of the dogs in each cohort with titres ≤0.1 IU/ml 360 days after vaccination. Between 20–40% of dogs overall had titres <0.5 IU/ml at the last time point. Excluding upper outliers, dogs in Zenzele with day 360 titres <0.5 IU/ml had a statistically significantly (Mann-Whitney test p<0.001) lower day 30 GMT (6.6 IU/ml, n = 38) compared to dogs with day 360 titres ≥0.5 IU/ml (23.6 IU/ml, n = 57); this is consistent with the correlations between time points discussed below.

#### Kinetics of titres in the vaccinated dogs

In Zenzele, titres declined rapidly between day 30 and 90, then gradually from day 90 (Figures 1–2). Log titres were closely correlated across all the time points, including between day 30 and 360 (excluding outliers correlation coefficient *r* = 0.55) and day 30 and the mean log titre for day 90, 180 and 360 (*r* = 0.72) (Table S16). Consequently, dogs with higher peak titres tended to have higher titres at the final time point. The model assuming a quadratic relationship between titre and time was a good fit, with predicted GMT for the day 30 (peak) and 360 titres congruent with the observed means (Table S17, model 1 and Table S18).

Peak (day 30) titres could not be extrapolated from the linear models for Bali, but the decline in titres between 180 and 360 in Kelusa was similar to Zenzele (Table S17, models 2–4). The GMT for Antiga declined only marginally with time (slope p = 0.4; Table S17, model 5), with a rate of decline less than quarter that of Zenzele and Kelusa. Overall, the predicted GMTs were comparable to the observed titres (Table S17, models 1–5 and Table S18).

The magnitude of any decline in titre as a consequence of extended storage time or freeze-thaw cycles was not great compared to normal background variation. This agrees with other studies evaluating the effect of storage time and freeze-thaw cycles on blood proteins [76], [77]. The decline in titre for 22 (88%) of the samples did not exceed normal inter-assay variation of two-fold or less [78].

#### Factors associated with variations in titre

When comparing the research cohorts, all models with time (points) had lower AICs than the null models except for Antiga when upper outliers were excluded, indicating that time after vaccination had an effect on titre. While there were no clear patterns across the cohorts between variations in titre and the covariates described under *Covariates* in the Materials and Methods, lactation and health status emerged as significant covariates.

For Zenzele, apart from lactation at vaccination, there were no statistically significant (p<0.05) associations of titre with age, gender, reproductive and health status, and body condition when accounting for all the time points (i.e. for models of the longitudinal data). Time and lactation were the only covariates retained in the quadratic model with the lowest AIC, where the negative effect of lactation was statistically significant (p≤0.02) (Table S20, model 1). When the response variable was restricted to peak (day 30) titres, titres again varied significantly with lactation (p≤0.02) (Table S20, model 2). Overall, the GMT of lactating dogs (∼6 IU/ml) was less than half that of males and non-lactating females. Clinical signs at the time of vaccination was also significant (p = 0.04) when the response variable was restricted to peak (day 30) titres but only when upper outliers were included in the model (Table S20, model 3). Those dogs with clinical signs at the time of vaccination had a GMT of 11 IU/ml, approximately half that of dogs without clinical signs (21 IU/ml). When body condition was dropped from the model, clinical signs at the time of vaccination was marginally statistically significant (p = 0.06) (Table S20, Note 4); the differences in factor levels (i.e. with and without clinical signs at vaccination) between the two models were comparable (9.5 IU/ml including and 8.0 IU/ml excluding body condition).

Lactation and health status at vaccination were also the main covariates of significance in the Bali villages. Lactation and pregnancy were excluded from the models for Kelusa (Table S21) given their small group sizes (Table S24) and these covariates were not retained in any of the models for Antiga (Table S22). However, a positive effect of lactation at vaccination was marginally statistically significant (p = 0.07) to statistically significant (p = 0.05) for Kelusa and Antiga combined when upper outliers were included in the model (Table S23, model 1 and Note 2).

When generalised dermatitis at the time of vaccination was included in the combined model, lactation was no longer significant (Table S23, model 2). Generalised dermatitis was statistically significant (p<0.02), although the GMT was only approximately 0.5 IU/ml less than the baseline (i.e. dogs not lactating without dermatitis GMT ∼2 IU/ml). Generalised dermatitis was retained in models for Kelusa, but it was not statistically significant (p = 0.15) (Table S21, model 1) even though 44 (37%) of dogs at the time of vaccination were affected (Table S24). However, generalised dermatitis at the time of vaccination was generally highly statistically significant for the Antiga cohort (p≤0.01) reducing the GMT by up to half (Table S22, models 2 and 3).

Convergence errors, regardless of fitting method, precluded full evaluation of the data set combining all three research cohorts.

#### Rabivet Supra 92

Of the 16 dogs vaccinated in December 2009 with Rabivet Supra 92 all had titres <0.5 IU/ml except for one dog at day 180 and two at day 360 sampling (Table S25).

#### Lagos bat virus assays

The samples were negative for Zenzele. All the samples were negative for Bali except for one vaccinated dog in Kelusa, with a 50% end-point titre of 1/64 upon initial testing and 1/32 upon re-testing.

#### Other diagnostics

Lymphocyte counts were significantly associated with body condition at the time of vaccination (p = 0.03) (Table S20, model 4), however there was no association with peak (day 30) titres (p>0.05). There were no associations between eosinophil counts and titres at any time point.

Almost all of the dogs had intestinal parasites, primarily *Ancylostoma spp.* (Table S26) [79], [80]. Consequently, there was insufficient variability to determine the effect of intestinal parasites on immune response to vaccination. One dog was positive for *Demodex spp.* on deep skin scrape.

## Discussion

The longitudinal, individual-level data from this study provides the most detailed serological data currently available for domestic dogs in rabies endemic areas, and provides valuable support for planning rabies vaccination programmes.

This study reinforces the importance of frequent and regular vaccination campaigns to ensure effective vaccination coverage is maintained. Dogs with lower peak titres had correspondingly lower titres at the end of the study, with titres <0.5 IU/ml at the last time point (day 360) for 20–40% of the dogs and <0.1 IU/ml for 3–8% of the dogs (Table S14); the implication being an increased susceptibility to natural exposure with time in the dogs with low titres [27], [34], [37], [40], [46]. Robust demographic data from these study populations indicates, two years after a pulse campaign which achieved 80% vaccination coverage, at least 20–45% vaccination coverage would remain [19], which is the critical threshold necessary to prevent rabies [24]. However, from our model predictions (Table S17), we speculate that a substantial proportion of the dogs remaining in Zenzele two years after vaccination may have titres <0.1 IU/ml, potentially dropping effective vaccination coverage to below the critical threshold. Models were constrained to two time points for the Bali cohorts, but predicted similar declines in the GMT for Kelusa.

The vast majority of the dogs seroconverted following vaccination (with a peak titre of ≥0.5 IU/ml), regardless of health status. However, there was considerable variation in titres at each time point for all the cohorts. Peak titres were not measured for the Bali cohorts, however day 180 titres were comparable to Zenzele, therefore it is likely that a similar proportion of dogs to Zenzele seroconverted following vaccination. Identification of risk factors associated with lower titres may promote targeted boostering to maintain vaccination coverage. Clinical conditions around the time of vaccination reduced the immune response to the vaccine in all the cohorts; in particular, generalised dermatitis provided a ‘visible marker’ for a reduced immune response, with practical implications for rabies control. While demodicosis was assumed to be an important cause of generalised dermatitis associated with immuno-suppression in Bali, the mostly negative skin scrapes suggests that dermatophytosis may be more likely, consistent with both the tropical climate and immuno-suppression [65], [66]. This warrants further investigation given that a substantial proportion of the dogs (37%–46% Table S24) were affected, potentially reducing the effectiveness of vaccination. Lactation at the time of vaccination in Zenzele and the Bali cohorts combined was significant statistically, however its biological significance is unclear. Lactation is associated with loss of body condition in all the research sites [19], consistent with immuno-suppression observed in Zenzele. The reason for the opposite effect in Bali cannot be readily explained [81], [82]. While this incongruity may warrant further investigation in larger study populations on balance lactating bitches should be vaccinated, with re-vaccination following weaning.

Our study demonstrated an advantage of community engagement and door-to-door vaccine programmes over the use of simple vaccination points. We consistently achieved vaccination coverage above 70% through door-to-door vaccine delivery, even in Bali where the majority of the dogs needed to be caught by net on successive occasions. Similar coverage was achieved across the rest of the island through door-to-door vaccine delivery in 2010 and 2011 [83]. This compares to a vaccination coverage of only 27% through the vaccination point in Zenzele and a very low vaccine uptake (5%) in Kelusa. The utility of vaccination points is likely to differ between locations according to local circumstances. Similar to other communities in Africa, Europe and central Asia where free-roaming dogs are handleable [11], [13], [84], [85], it is likely that the majority of the dogs in Zenzele could have been delivered to the vaccination point by their owners, and the low vaccination coverage was probably the result of inadequate advertising [86] and limited operating hours during a work/school day. Vaccine uptake in Kelusa was, in part, affected by community awareness of the research vaccination program, however the majority of the dogs could not be handled by their owners or the vaccinators, thus necessitating restraint by net [83]. The reasons for the difference in handleability between locations are unclear. Restraint by net is more stressful to the dog, time consuming and costly than by leash and muzzle. In order to improve welfare, facilitate more cost-effective and efficient delivery of vaccines (and other prophylactics), and improve evaluation of the dogs in Bali and similar communities, extending our studies to evaluate the differences in husbandry, environment and other factors influencing the temperament of the dogs in the sites is warranted.

This research has generated valuable data that may contribute to rabies control, including through improving epidemiological models. However, understanding variation between dogs in titres measured from field studies is challenging. Some covariates that may impact on titres, such as lactation and health status, are measurable, whereas others such as genetics and stress are harder to assess in real time. Further evaluation of factors associated with variation in immunity over time since vaccination, including both serological responses and direct assessment of CMI, and recording vaccine failures is warranted and may require larger populations studied and over longer time periods.

### Conclusion

This study demonstrates that the vast majority of free-roaming dogs, in two regions of Africa and Asia where rabies is endemic, seroconverted to rabies vaccine regardless of health status, producing titres that exceeded 0.5 IU/ml, the level considered necessary to protect against rabies. Declines in vaccination coverage following a vaccination campaign occur through mortality/emigration of vaccinated dogs and birth/immigration of unvaccinated, susceptible dogs. Robust demographic data from the study populations show that two years after vaccinating at least 70% of dogs during a pulse vaccination campaign, vaccination coverage remained within 20–45% [19], the range necessary to control rabies (Hampson 2009). However, our serological data indicates that dogs with lower peak (day 30) titres had correspondingly lower end point (day 360) titres. We speculate that a proportion of vaccinated dogs remaining in the study populations after two years will probably have titres below the approximate threshold for protection (<0.1 IU/ml) thus dropping effective vaccination coverage to below the critical threshold (of 20–45%). This emphasizes the importance of re-vaccinating within two years. Vaccination of all dogs during annual campaigns is therefore recommended as the most effective means of ensuring that individual immunity and population coverage are both maintained at sufficient levels to control rabies.

## Supporting Information

Figure S1 a–c Population age structure.
(DOCX)

Table S1 Summary of the study methodology.
(DOCX)

Table S2 Summary of vaccination coverage.
(DOCX)

Table S3 The number of dogs in the research cohorts and the number of unvaccinated controls in Bali that were blood sampled at each time point.
(DOCX)

Table S4 Age-specific life expectancies (see Figures S1a–c).
(DOCX)

Table S5 Characteristics of the dogs in Bali (in January 2010) that were not vaccinated.
(DOCX)

Table S6 Description of the dogs in Zenzele with baseline (day 0) titres ≥0.5 IU/ml.
(DOCX)

Table S7 The distribution of the titres of the unvaccinated controls in Bali with titres ≥0.5 IU/ml.
(DOCX)

Table S8 The gender of the unvaccinated controls in Bali with titres ≥0.5 IU/ml.
(DOCX)

Table S9 The ages of the unvaccinated controls in Bali with titres ≥0.5 IU/ml.
(DOCX)

Table S10 Summary of the titres of the unvaccinated controls in Bali.
(DOCX)

Table S11 Summary of titres from the dogs in Zenzele necessary to assess the inadvertent inclusion of dogs vaccinated by the Department of Agriculture in October 2009 in the research cohort.
(DOCX)

Table S12 Summary of the titres of the dogs in the Zenzele research cohort present May 2006 and those that arrived into the population after May 2006.
(DOCX)

Table S13 Characteristics (at vaccination) of the dogs in the Zenzele research and DoA cohorts with peak (day 30) titres <0.5 IU/ml.
(DOCX)

Table S14 Summary of the day 180 and 360 titres in the research cohorts [vaccinated dogs].
(DOCX)

Table S15 Characteristics (at vaccination) of the dogs in the research cohorts with day 360 titres ≤0.1 IU/ml.
(DOCX)

Table S16 Correlation coefficients for the dogs in the Zenzele research cohort that were blood sampled at every time point (see Figure 1).
(DOCX)

Table S17 Models restricted to the natural log of the titre as the response variable and time as the covariate for the research cohorts.
(DOCX)

Table S18 Observed and predicted geometric mean titres for each time point.
(DOCX)

Table S19 Description of the covariates in the models detailed under *Statistical methods* in the Materials and Methods (see Text S2 and Tables S20, S21, S22, S23).
(DOCX)

Table S20 Zenzele linear mixed effects and analysis of variance model outputs.
(DOCX)

Table S21 Kelusa linear mixed effects model outputs.
(DOCX)

Table S22 Antiga linear mixed effects model outputs.
(DOCX)

Table S23 Bali linear mixed effects model outputs.
(DOCX)

Table S24 Contingency tables for the covariates in the models detailed under *Statistical methods* in the Materials and Methods (see Tables S20, S21, S22, S23).
(DOCX)

Table S25 Dogs vaccinated by the Department of Livestock in Kelusa with Rabivet Supra 92.
(DOCX)

Table S26 The number of dogs in Zenzele with intestinal parasites on day 0.
(DOCX)

Text S1 Sample selection and sampling technique for deep skin scrapes.
(DOCX)

Text S2 Description of the covariates in the models detailed under *Statistical methods* in the Materials and Methods (see Table S19).
(DOCX)

Text S3 Sample selection for faecal analysis (see Table S26).
(DOCX)
