# Supplementary material for: Achieving Population-Level Immunity to Rabies in Free-Roaming Dogs in Africa and Asia
Source: PLoS Negl Trop Dis. 2014 Nov 13;8(11):e3160. doi: 10.1371/journal.pntd.0003160 (PMC4230884; doi:10.1371/journal.pntd.0003160)
Supplement: Figure S1 — a–c Population age structure. (DOCX) [file pntd.0003160.s001.docx]

Figure S1a


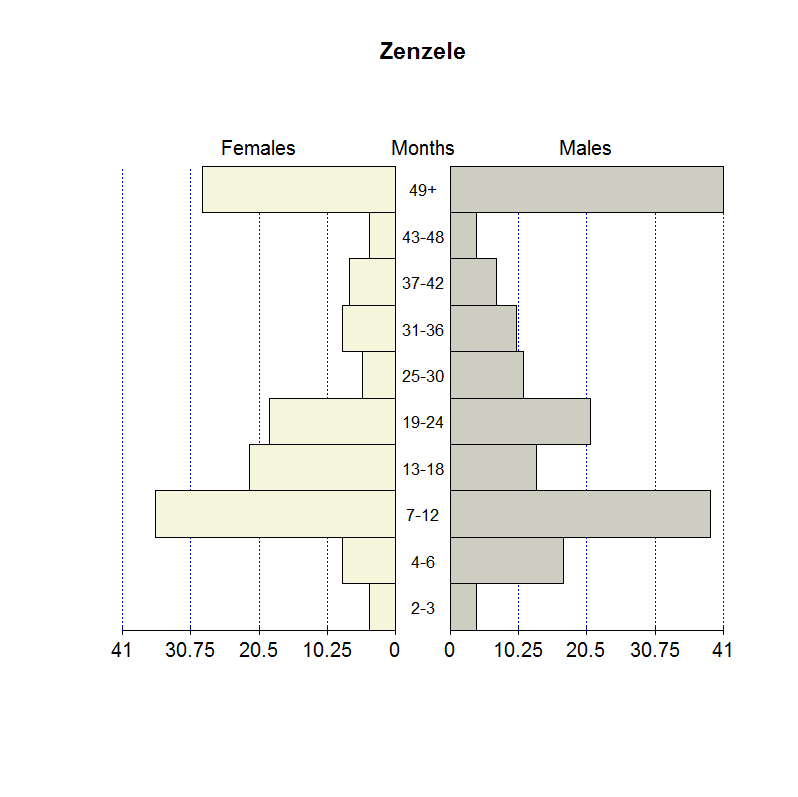


Figure S1b


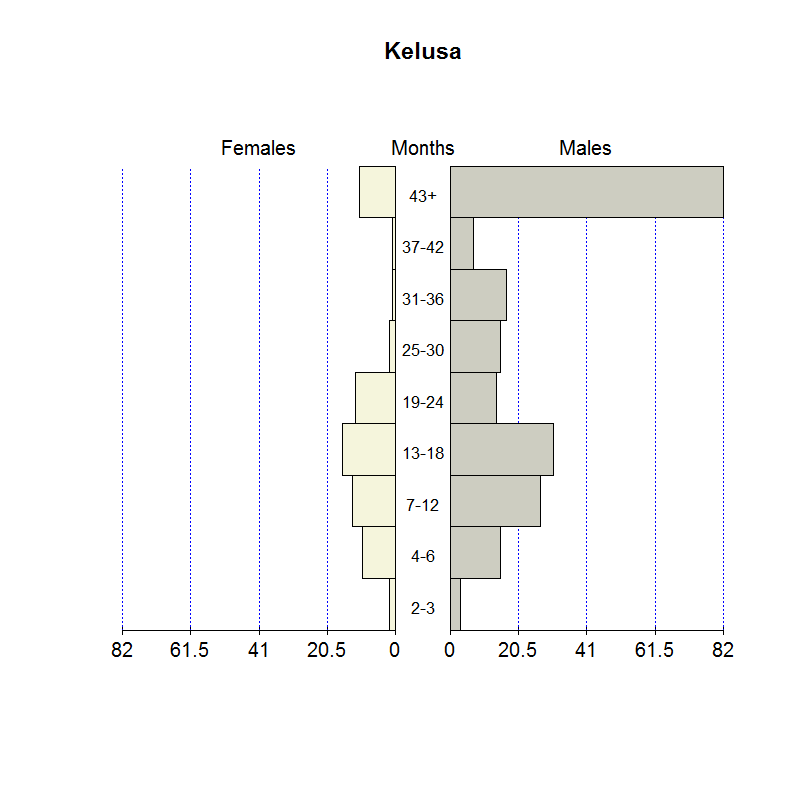


Figure S1c


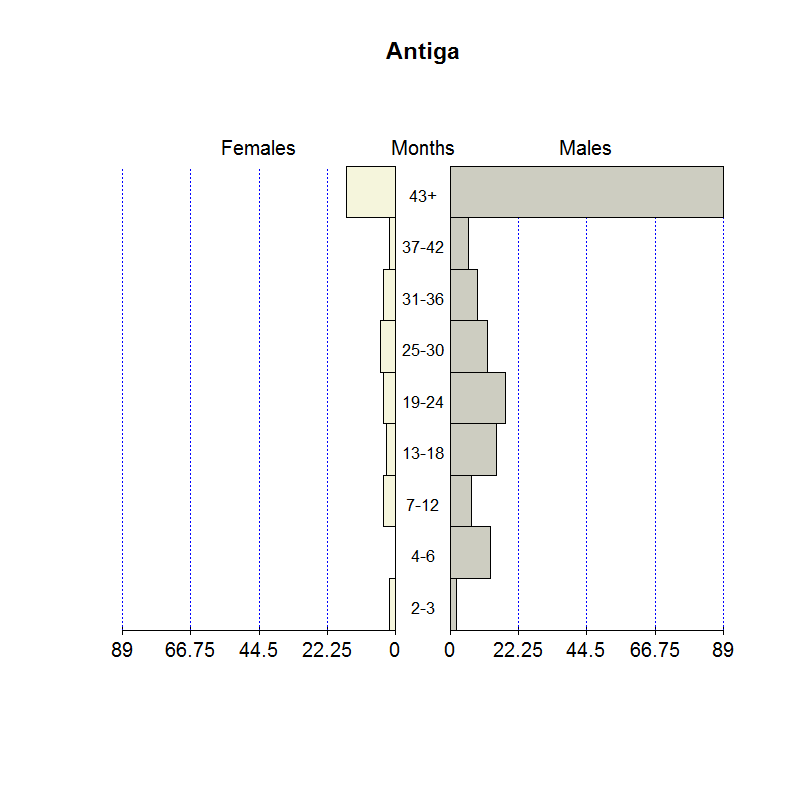


Figs. S1a-c. Population age structure is determined from the age distribution at the last time point of the (larger ecological) study period, which consists of the largest fraction of adults with known ages. Most dogs in their 42nd month of life or less had been observed as a pup or juvenile during the study period, so their true age was known. Additionally, several (Zenzele n= 9, Kelusa n= 9 and Antiga n=3) litters were born during the last time point. Reproduced from Morters *et al*. 2014 [[1](#_ENREF_1)]. The x-axis shows the number of individuals in each age class and the y-axis shows the month of life. For example, dogs in age class 2-3 are in their second or third month of life.

References

1. Morters MK, McKinley TJ, Restif O, Conlan AJK, Cleaveland S, et al. (2014) The demography of free-roaming dog populations and applications to disease and population control. Journal of Applied Ecology 51: 1096-1106.
