# Supplementary material for: Achieving Population-Level Immunity to Rabies in Free-Roaming Dogs in Africa and Asia
Source: PLoS Negl Trop Dis. 2014 Nov 13;8(11):e3160. doi: 10.1371/journal.pntd.0003160 (PMC4230884; doi:10.1371/journal.pntd.0003160)
Supplement: Table S1 — Summary of the study methodology. (DOCX) [file pntd.0003160.s002.docx]

Table S1 Summary of the study methodology

ᶧ upper outliers were those dogs in Zenzele (n=7) with histories and post-vaccinal titres suggestive of vaccination with Rabisin by the DoA through a vaccination point in May 2006 or undertaken independently by the owner

ꜗ upper outliers were those dogs in Kelusa and Antiga (n=4 and n=15 respectively) with histories and post-vaccinal titres suggestive of vaccination undertaken independently by the owner or as part of vaccination campaigns outside of Kelusa and Antiga
