# Supplementary material for: Achieving Population-Level Immunity to Rabies in Free-Roaming Dogs in Africa and Asia
Source: PLoS Negl Trop Dis. 2014 Nov 13;8(11):e3160. doi: 10.1371/journal.pntd.0003160 (PMC4230884; doi:10.1371/journal.pntd.0003160)
Supplement: Table S2 — Summary of vaccination coverage. (DOCX) [file pntd.0003160.s003.docx]

Table S2 Summary of vaccination coverage

| * There were an additional four households with at least one dog; three of the households owned mostly greyhounds that were confined to the yard  ᶧ There was one household where the details of the number of dogs owned was not available  ꜗ In Kelusa 146 dogs and in Antiga 124 dogs were caught for blood sampling at both time points (including vaccinated and unvaccinated dogs)  ‡ see Table S25 |
| --- |

Note: puppies born in the study area within ~12 weeks prior to vaccination/sampling but not observed during the vaccination/sampling period were assumed to have been lost prior to the commencement of the vaccination / sampling
