# Supplementary material for: Achieving Population-Level Immunity to Rabies in Free-Roaming Dogs in Africa and Asia
Source: PLoS Negl Trop Dis. 2014 Nov 13;8(11):e3160. doi: 10.1371/journal.pntd.0003160 (PMC4230884; doi:10.1371/journal.pntd.0003160)
Supplement: Table S3 — The number of dogs in the research cohorts and the number of unvaccinated controls in Bali that were blood sampled at each time point. (DOCX) [file pntd.0003160.s004.docx]

Table S3 The number of dogs in the research cohorts and the number of unvaccinated controls in Bali that were blood sampled at each time point

* day 0 immediately prior to vaccination for the research cohort

Note 1: of the 97 dogs in Zenzele that were vaccinated by the DoA in October 2009, 60 were blood sampled 8-10 days after vaccination, and 77 were sampled 30 days, 65 were sampled 90 days, 64 were sampled 180 days, and 47 dogs were sampled 360 days after vaccination

Note 2: in addition to the dogs recorded in Table S3:

- Kelusa day 180: 11 dogs vaccinated by the DoL Dec-09 sampled, 1 dog taken by the owner to an NGO for vaccination sampled, 1 dog sampled but its vaccination history was uncertain, 1 missing sample; there were an additional 32 puppies <6-8 weeks of age present in the study area during the sampling period
- Kelusa day 360: 12 dogs vaccinated by the DoL Dec-09 sampled, 1 dog taken by the owner to an NGO for vaccination sampled, 1 dog sampled but its vaccination history was uncertain, 33 dogs vaccinated only (mostly puppies <6-8 weeks of age)
- Antiga day 180: 3 dogs vaccinated by the DoL Apr-10 sampled, 1 dog taken by the owner to an NGO for vaccination sampled, 1 dog sampled but its vaccination history was unknown, 2 missing samples; there were an additional 19 puppies <6-8 weeks of age present in the study area during the sampling period
- Antiga day 360: 3 dogs vaccinated by the DoL Apr-10 sampled, 9 dogs in Ketug inadvertently vaccinated by the NGO involved in the island wide vaccination campaign sampled ~30 days after vaccinated (titres ranged from 2-181 IU/ml), 2 dogs taken by the owner to an NGO for vaccination sampled, 13 dogs vaccinated only (mostly puppies <6-8 weeks of age)
