# Supplementary material for: Achieving Population-Level Immunity to Rabies in Free-Roaming Dogs in Africa and Asia
Source: PLoS Negl Trop Dis. 2014 Nov 13;8(11):e3160. doi: 10.1371/journal.pntd.0003160 (PMC4230884; doi:10.1371/journal.pntd.0003160)
Supplement: Table S4 — Age-specific life expectancies (see Figures S1a–c). (DOCX) [file pntd.0003160.s005.docx]

Table S4 Age-specific life expectancies for the entire (ecological study) research population derived from the observed ages at the last time point of the study period (see Figures S1a-c)
