# Supplementary material for: Achieving Population-Level Immunity to Rabies in Free-Roaming Dogs in Africa and Asia
Source: PLoS Negl Trop Dis. 2014 Nov 13;8(11):e3160. doi: 10.1371/journal.pntd.0003160 (PMC4230884; doi:10.1371/journal.pntd.0003160)
Supplement: Table S5 — Characteristics of the dogs in Bali (in January 2010) that were not vaccinated. (DOCX) [file pntd.0003160.s006.docx]

Table S5 Characteristics of the dogs in Bali (in January 2010) that were not vaccinated on day 0;

the number of dogs that could not be caught for vaccination is shown in brackets

* dogs in age class 1-6 months are between birth [start of their 1st month of life] and ~26 weeks of age [end of their 6th month of life], and so on; mature adults are mostly dogs first observed as adults at the start of the (larger ecological) study period (i.e. from March 2008), but also dogs first observed as adults after the start of this study period, therefore the exact age of these dogs could not be determined by direct observation [Note: dogs in age classes 1-6, 7-12 and 13-36 months were observed as pups or juveniles from March 2008, so their true age was known]

ᶧ in addition, consent for vaccination was declined for 3 dogs including 1 male in its 20th month of life, 1 mature adult female, and 1 dog of unknown gender and age

ꜗ in addition, consent for vaccination was declined for at least 7 dogs including 5 mature adult males, 1 male in its 13th month of life, and 1 dog of unknown gender and age
