# Supplementary material for: Achieving Population-Level Immunity to Rabies in Free-Roaming Dogs in Africa and Asia
Source: PLoS Negl Trop Dis. 2014 Nov 13;8(11):e3160. doi: 10.1371/journal.pntd.0003160 (PMC4230884; doi:10.1371/journal.pntd.0003160)
Supplement: Table S6 — Description of the dogs in Zenzele with baseline (day 0) titres ≥0.5 IU/ml. (DOCX) [file pntd.0003160.s007.docx]

Table S6 Description of the dogs in Zenzele with baseline (day 0) titres ≥0.5 IU/ml

* ≥36th month of life in February 2010 (at vaccination)
