# Supplementary material for: Achieving Population-Level Immunity to Rabies in Free-Roaming Dogs in Africa and Asia
Source: PLoS Negl Trop Dis. 2014 Nov 13;8(11):e3160. doi: 10.1371/journal.pntd.0003160 (PMC4230884; doi:10.1371/journal.pntd.0003160)
Supplement: Table S7 — The distribution of the titres of the unvaccinated controls in Bali with titres ≥0.5 IU/ml. (DOCX) [file pntd.0003160.s008.docx]

Table S7 The distribution of the titres of the unvaccinated controls in Bali with titres ≥0.5 IU/ml

ᶧ one dog was sampled at both time points [day 180 titre = 1 IU/ml, day 360 titre = 0.5 IU/ml]
