# Supplementary material for: Achieving Population-Level Immunity to Rabies in Free-Roaming Dogs in Africa and Asia
Source: PLoS Negl Trop Dis. 2014 Nov 13;8(11):e3160. doi: 10.1371/journal.pntd.0003160 (PMC4230884; doi:10.1371/journal.pntd.0003160)
Supplement: Table S10 — Summary of the titres of the unvaccinated controls in Bali. (DOCX) [file pntd.0003160.s011.docx]

Table S10 Summary of the titres of the unvaccinated controls in Bali

* 1 dog with a titre 2.83 IU/ml was acquired as a pup from outside the survey area (Ubud) and may have been vaccinated in Ubud; the Mann-Whitney test p-value remains <0.001

after removing this dog from the analysis. The Mann-Whitney test was used to compare the mean titres between unvaccinated dogs in Kelusa and Antiga for the same time

points (see *Statistical methods* in the Methods and materials)
