# Supplementary material for: Achieving Population-Level Immunity to Rabies in Free-Roaming Dogs in Africa and Asia
Source: PLoS Negl Trop Dis. 2014 Nov 13;8(11):e3160. doi: 10.1371/journal.pntd.0003160 (PMC4230884; doi:10.1371/journal.pntd.0003160)
Supplement: Table S11 — Summary of titres from the dogs in Zenzele necessary to assess the inadvertent inclusion of dogs vaccinated by the Department of Agriculture in October 2009 in the research cohort. (DOCX) [file pntd.0003160.s012.docx]

Table S11 Summary of titres from the dogs in Zenzele necessary to assess the inadvertent inclusion of dogs vaccinated by the Department of Agriculture in October 2009 in the research cohort
