# Supplementary material for: Achieving Population-Level Immunity to Rabies in Free-Roaming Dogs in Africa and Asia
Source: PLoS Negl Trop Dis. 2014 Nov 13;8(11):e3160. doi: 10.1371/journal.pntd.0003160 (PMC4230884; doi:10.1371/journal.pntd.0003160)
Supplement: Table S12 — Summary of the titres of the dogs in the Zenzele research cohort present May 2006 and those that arrived into the population after May 2006. (DOCX) [file pntd.0003160.s013.docx]

Table S12 Summary of the titres of the dogs in the Zenzele research cohort present May 2006 and those that arrived into the population after May 2006

(excluding 8 dogs whose presence in May 2006 was unknown; including upper outliers)

ᶧ 2 dogs with titres of 0.5 IU/ml and 1 dog with a titre of 1 IU/ml; * 2 dogs with titres of 0.71 IU/ml. The Mann-Whitney test was used to compare the mean titres between dogs present in Zenzele

in May 2006 and those that arrived into the population after May 2006 (see *Statistical methods* in the Methods and materials)
