# Supplementary material for: Achieving Population-Level Immunity to Rabies in Free-Roaming Dogs in Africa and Asia
Source: PLoS Negl Trop Dis. 2014 Nov 13;8(11):e3160. doi: 10.1371/journal.pntd.0003160 (PMC4230884; doi:10.1371/journal.pntd.0003160)
Supplement: Table S13 — Characteristics (at vaccination) of the dogs in the Zenzele research and DoA cohorts with peak (day 30) titres <0.5 IU/ml. (DOCX) [file pntd.0003160.s014.docx]

Table S13 Characteristics (at vaccination) of the dogs in the Zenzele research and DoA cohorts with peak (day 30) titres <0.5 IU/ml

* ≥36th month of life in February 2010 (at vaccination); ˚ ≥32nd month of life in October 2009 (at vaccination)

ᶧ peak titre missing but titres <0.5 IU/ml at all other time points; key: me = missing entry, NA = not applicable
