# Supplementary material for: Achieving Population-Level Immunity to Rabies in Free-Roaming Dogs in Africa and Asia
Source: PLoS Negl Trop Dis. 2014 Nov 13;8(11):e3160. doi: 10.1371/journal.pntd.0003160 (PMC4230884; doi:10.1371/journal.pntd.0003160)
Supplement: Table S14 — Summary of the day 180 and 360 titres in the research cohorts [vaccinated dogs]. (DOCX) [file pntd.0003160.s015.docx]

Table S14 Summary of the day 180 and 360 titres in the research cohorts [vaccinated dogs] (excluding upper outliers)

* 16 dogs had titres of 1 IU/ml, 16 dogs had titres of 1.41 IU/ml, and 16 dogs had titres of 2 IU/ml. The Mann-Whitney test was used to compare the mean titres between vaccinated dogs in Zenzele, Kelusa and Antiga for the same time points (see *Statistical methods* in the Methods and materials)
