# Supplementary material for: Achieving Population-Level Immunity to Rabies in Free-Roaming Dogs in Africa and Asia
Source: PLoS Negl Trop Dis. 2014 Nov 13;8(11):e3160. doi: 10.1371/journal.pntd.0003160 (PMC4230884; doi:10.1371/journal.pntd.0003160)
Supplement: Table S15 — Characteristics (at vaccination) of the dogs in the research cohorts with day 360 titres ≤0.1 IU/ml. (DOCX) [file pntd.0003160.s016.docx]

Table S15 Characteristics (at vaccination) of the dogs in the research cohorts with day 360 titres ≤0.1 IU/ml

* ≥36th month of life in February 2010 in Zenzele for the research cohort, 32nd month of life in October 2009 in Zenzele for the DoA cohort, 34th month in January 2010 in Kelusa, and 33rd month in January 2010 in Antiga (at vaccination); ᶧ month of life is in this range; key: me = missing entry, NA = not applicable
