# Supplementary material for: Achieving Population-Level Immunity to Rabies in Free-Roaming Dogs in Africa and Asia
Source: PLoS Negl Trop Dis. 2014 Nov 13;8(11):e3160. doi: 10.1371/journal.pntd.0003160 (PMC4230884; doi:10.1371/journal.pntd.0003160)
Supplement: Table S16 — Correlation coefficients for the dogs in the Zenzele research cohort that were blood sampled at every time point (see Figure 1). (DOCX) [file pntd.0003160.s017.docx]

Table S16 Correlation coefficients for the dogs in the Zenzele research cohort that were blood sampled at every

time point (i.e. day 30, 90, 180 and 360) (see Figure 1)
