# Supplementary material for: Achieving Population-Level Immunity to Rabies in Free-Roaming Dogs in Africa and Asia
Source: PLoS Negl Trop Dis. 2014 Nov 13;8(11):e3160. doi: 10.1371/journal.pntd.0003160 (PMC4230884; doi:10.1371/journal.pntd.0003160)
Supplement: Table S17 — Models restricted to the natural log of the titre as the response variable and time as the covariate for the research cohorts. (DOCX) [file pntd.0003160.s018.docx]

Table S17 Models restricted to the natural log of the titre as the response variable and time as the covariate for the research cohorts; natural logs are shown in the tables

Table S17 continued

Note: in addition to excluding the 15 dogs with day 180 titres ≥11.3 IU/ml, excluding the 3 dogs with day 360 titres of 11.3 IU/ml results in observations = 258, intercept = 0.0907 p= 0.518, time = -0.0006 p = 0.213

* confidence intervals could not be obtained on the variance-covariance components using the intervals function with lme{nlme}; therefore, estimates of the confidence intervals were derived using the lme4 package with lmer, profile and confint functions
