# Supplementary material for: Achieving Population-Level Immunity to Rabies in Free-Roaming Dogs in Africa and Asia
Source: PLoS Negl Trop Dis. 2014 Nov 13;8(11):e3160. doi: 10.1371/journal.pntd.0003160 (PMC4230884; doi:10.1371/journal.pntd.0003160)
Supplement: Table S18 — Observed and predicted geometric mean titres for each time point. (DOCX) [file pntd.0003160.s019.docx]

Table S18 Observed and predicted geometric mean titres for each time point; predictions are derived from the models restricted to natural log of the titre as the response variable and time as the covariate (Table S17)
