# Supplementary material for: Achieving Population-Level Immunity to Rabies in Free-Roaming Dogs in Africa and Asia
Source: PLoS Negl Trop Dis. 2014 Nov 13;8(11):e3160. doi: 10.1371/journal.pntd.0003160 (PMC4230884; doi:10.1371/journal.pntd.0003160)
Supplement: Table S19 — Description of the covariates in the models detailed under Statistical methods in the Materials and Methods (see Text S2 and Tables S20, S21, S22, S23). (DOCX) [file pntd.0003160.s020.docx]

Table S19 Description of the covariates in the models detailed under *Statistical methods* in the Methods and materials (see Stext2 and Tables S20-S23)

| covariate description, factor levels included in the analysis, and period of evaluation | methods of evaluation |
| --- | --- |
| house and dog identification  gender (male or female) | *What is the dog’s name?*  direct observation  direct observation |
| at day 0 (vaccination):  age class  (month of life: 1-6, 7-12, 13-36, mature adult)*  sterilisation status (yes=1 / no=0)  pregnancy or lactation (yes=1 / no=0) | *How old is this dog* or *When did you get this dog?*  *How old was the dog when you got it?*  direct observation  *Has your (female) dog been sterilised* or *Has she had an operation to stop her having puppies?*  direct observation of male dogs  direct observation; whelping and vaccination dates |
| Zenzele Jan-10 - Mar-10; Bali Dec-09 - Feb-10:  body condition minimum and maximum  (thin [1-3] or fat [4+]; or  2, 3, 2-3, 4, 5, 5+ [also 6+ in Zenzele]) | direct observation |
| Zenzele Jan-10/Feb-10; Bali Dec-09/Jan-10:  clinical signs associated with serious illnessᶧ and generalised dermatitis  (present=1 / not present=0)  generalised dermatitis as a separate category (present=1 / not present=0) | direct observation and owner reporting based on a set of pictures  In reference to the pictures:  *Has this dog had any of these problems the past 7 days?*  *Has this dog had any other problems the past 7 days?*  *Has this dog had any of these problems since our last visit?*  *Has this dog had any other problems since our last visit?* |
| Bali Dec-09/Jan-10:  protein intake  (never or rarely=0 /  more frequent than never or rarely=1) | owner reporting based on detailed discussions |

* see Table S5 for the definition

ᶧ including, but not limited to, vomiting; diarrhoea; dysuria; dyschezia; constipation; swollen stomach; drooling/salivation; dehydration; increase or decrease in eating or drinking; recent weight loss; jaundiced, pale, hyperaemic or cyanotic mucous membranes; coughing; dyspnoea or tachypnoea; ataxia; lethargy/depression; recumbency; severe injury
