# Supplementary material for: Achieving Population-Level Immunity to Rabies in Free-Roaming Dogs in Africa and Asia
Source: PLoS Negl Trop Dis. 2014 Nov 13;8(11):e3160. doi: 10.1371/journal.pntd.0003160 (PMC4230884; doi:10.1371/journal.pntd.0003160)
Supplement: Table S20 — Zenzele linear mixed effects and analysis of variance model outputs. (DOCX) [file pntd.0003160.s021.docx]

Table S20 Zenzele models; the full range of models were tested with natural log of the titre as the response variable and the covariates described under *Covariates* in the Methods and materials; all models with the lowest AIC retained time as the only covariate (i.e. for the quadratic models, see Table S17) or were null (i.e. for the models fitted to a single time point) with the exception of the models shown below; natural logs are shown in the tables

Table S20 Zenzele models continued

Table S20 Zenzele models continued

Note 1: Models 1 and 2 treat the following dogs as not lactating at the time of vaccination: (i) three dogs that whelped in November 2009 and still had at least one of their pups present at vaccination (February 2010) but these pups were probably fully weaned (at vaccination), (ii) one dog that whelped in February 2010 but the survival/number of pups present at vaccination was uncertain, and (iii) two dogs that whelped in December 2009 and still had at least one of their pups present at vaccination but it was uncertain if the pups were fully weaned [treating these two dogs as lactating at vaccination in the quadratic model with upper outliers intercept = 2.6424, lactating = -0.7435 p = 0.022, and without upper outliers intercept = 2.5504, lactating = -0.8385 p = 0.010; and in the single time point model with upper outliers intercept = 2.8485, lactating = -0.7931 p = 0.059, and without upper outliers intercept = 2.7583, lactating = -0.9359 p = 0.025]

Note 2: One additional linear model, fitted to four time points (day 30, 90 180 and 360) with the intercept adjusted to day 30, with the lowest AIC retained covariates other than time. The model included natural log of the titre as the response variable and time, age, gender, pregnancy, lactation and body condition as covariates. The results were similar to Model 1 (i.e. the larger data set). Apart from time, the model with the lowest AIC retained lactation at the time of vaccination only. Treating the two dogs described under Note 1(iii) as not lactating at vaccination, with upper outliers observations = 449, intercept = 2.6664, lactating = -1.0848 p = 0.009, and without upper outliers observations = 432, intercept = 2.5599, lactating = -1.0097 p = 0.012. Treating the two dogs described under Note 1(iii) as lactating at vaccination, with upper outliers observations = 449, intercept = 2.6686, lactating = -0.8981 p = 0.016, and without upper outliers observations = 432, intercept = 2.5697, lactating = -1.0511 p = 0.006.

Note 3: One additional linear model, fitted to a single time point (day 30) with the lowest AIC, retained covariates. The model included natural log of the titre as the response variable and age, gender, pregnancy, lactation and body condition as covariates. The results were similar to Model 2 (i.e. the larger data set). The model with the lowest AIC retained lactation at the time of vaccination only. Treating the two dogs described under Note 1(iii) as not lactating at vaccination, with upper outliers observations = 144, intercept = 2.8825, lactating = -1.4486 p = 0.008, and without upper outliers observations = 138, intercept = 2.7756, lactating = -1.3417 p = 0.011. Treating the two dogs described under Note 1(iii) as lactating at vaccination, with upper outliers observations = 144, intercept = 2.8712, lactating = -0.9963 p = 0.043, and without upper outliers observations = 138, intercept = 2.7783, lactating = -1.2342 p = 0.013.

Note 4: One additional linear model, fitted to a single time point (day 30) with the lowest AIC, retained covariates. The model included natural log of the titre as the response variable and age, pregnancy, lactation and clinical signs as covariates. The results were similar to Model 3 (i.e. the smaller data set). The model with the lowest AIC retained clinical signs at the time of vaccination only. With upper outliers observations = 122, intercept = 2.9553, with clinical signs =

-0.5365 **p = 0.063**. Including gender in the model, with upper outliers and treating the two dogs described under Note 1(iii) as lactating intercept = 2.9553, with clinical signs = -0.5365 **p = 0.063**; and, treating the two dogs described under Note 1(iii) as not lactating intercept = 3.2086, with clinical signs = -0.5583 p = 0.054, lactating = -0.8403 p = 0.14, male = -0.4230 p = 0.13. Without upper outliers all the models were null.
