# Supplementary material for: Achieving Population-Level Immunity to Rabies in Free-Roaming Dogs in Africa and Asia
Source: PLoS Negl Trop Dis. 2014 Nov 13;8(11):e3160. doi: 10.1371/journal.pntd.0003160 (PMC4230884; doi:10.1371/journal.pntd.0003160)
Supplement: Table S21 — Kelusa linear mixed effects model outputs. (DOCX) [file pntd.0003160.s022.docx]

Table S21 Kelusa models; the full range of models were tested with natural log of the titre as the response variable and the covariates described under *Covariates* in the Methods and materials; all models with the lowest AIC retained time as the only covariate (see Table S17) with the exception of the models shown below; natural logs are shown in the tables

Note 1: Two dogs with incomplete observational data for generalised dermatitis during December 2009 and January 2010 but with chronic, generalised dermatitis diagnosed by direct observation prior to December 2009 and after January 2010 almost certainly had generalised dermatitis when vaccinated. Model 1 treats these dogs as having generalised dermatitis at vaccination.

Note 2: One additional linear model, fitted to two points (day 180 and 360) with the intercept adjusted to day 30, with the lowest AIC retained covariates other than time. The model included natural log of the titre as the response variable and time, age, gender, protein intake, sterilisation, body condition, clinical signs and generalised dermatitis as covariates. The results were similar to Model 1 (i.e. the larger data set). Apart from time, the model with the lowest AIC excluded upper outliers and retained generalised dermatitis at the time of vaccination only [observations = 183, intercept = 0.8961, with generalised dermatitis = -0.3358 p = 0.151].
