# Supplementary material for: Achieving Population-Level Immunity to Rabies in Free-Roaming Dogs in Africa and Asia
Source: PLoS Negl Trop Dis. 2014 Nov 13;8(11):e3160. doi: 10.1371/journal.pntd.0003160 (PMC4230884; doi:10.1371/journal.pntd.0003160)
Supplement: Table S23 — Bali linear mixed effects model outputs. (DOCX) [file pntd.0003160.s024.docx]

Table S23 Bali models; the full range of models were tested with natural log of the titre as the response variable and the covariates described under *Covariates* in the Methods and materials; all models with the lowest AIC retained time as the only covariate (see Table S17) with the exception of the models shown below; natural logs are shown in the tables

* confidence intervals could not be obtained on the variance-covariance components using the intervals function with lme{nlme}; therefore, estimates of the confidence intervals were derived using the lme4 package with lmer, profile and confint functions

ᶧ possible identifiability issues precluded estimates of the confidence intervals using the intervals function with lme{nlme};

rather estimates of the confidence intervals were derived using the lme4 package with the lmer, profile and confint functions

Table S23 Bali models continued

Note 1: Two dogs in Antiga had incomplete observational data for lactation at the time of vaccination but were almost certainly lactating when vaccinated (January 2010) based on the presence of puppies at vaccination and the bitches’ whelping and vaccination dates. Models 1 and 2 treat these dogs as lactating at vaccination.

Note 2: Models 1 and 2 treat the following dogs as not lactating at the time of vaccination: (i) one dog in Antiga that did not whelp at the owner’s house and, consequently, the survival/number of pups present at vaccination was uncertain, (ii) one dog in Kelusa that whelped in October 2009 and still had at least one pup present January 2010 but the pups were probably fully weaned at vaccination, and (iii) one dog in Kelusa that whelped in January but the vaccination date of the bitch was different to the puppies [treating this dog as lactating at vaccination in Model 1 (with upper outliers), given the likely scenario that she was vaccinated immediately after whelping and vaccination of the puppies was delayed until they were bigger, intercept = 0.4919, lactating = 0.7545 **p = 0.05**; treating this dog as lactating at vaccination in Model 2 (with upper outliers) intercept = 0.7484, lactating = 0.6726 p = 0.11, with generalised dermatitis = -0.3547 p = 0.019].

Note 3: One additional linear model, fitted to two time points (day 180 and 360) with the intercept adjusted to day 30, with the lowest AIC retained covariates other than time. The model included natural log of the titre as the response variable and time, age, gender, pregnancy and lactation (with the same inclusion/exclusion criteria for lactation as Models 1 and 2, see Notes 1 and 2 above). Treating the dog in Kelusa vaccinated ~ 2 weeks before her puppies (as per Note 2(iii)) as lactating at the time of vaccination, apart from time the model with the lowest AIC excluded upper outliers and retained lactation at vaccination only [observations = 549, intercept = 0.3020, lactating = 0.5822 p = 0.13].

Note 4: Two dogs in Kelusa (as per Table S21, Note 1) and three dogs in Antiga (as per Table S22, Note 1) with incomplete observational data for generalised dermatitis during December 2009 and January 2010 but diagnosed with chronic, generalised dermatitis by direct observation prior to December 2009 and after January 2010 almost certainly had generalised dermatitis when vaccinated. Model 2 treats these dogs as having generalised dermatitis at the time of vaccination.

Note 5: One additional linear model, fitted to two time points (day 180 and 360) with the intercept adjusted to day 30, with the lowest AIC retained covariates other than time. The model included natural log of the titre as the response variable and time, age, gender, pregnancy, lactation and body condition as covariates (with the same inclusion/exclusion criteria for lactation as Models 1 and 2, see Notes 1 and 2 above). The results were similar to Model 1 (i.e. the larger data set). Treating the dog in Kelusa vaccinated ~ 2 weeks before her puppies (as per Note 2(iii)) as not lactating at the time of vaccination, apart from time the model with the lowest AIC included upper outliers and retained lactation at vaccination only [observations = 437, intercept = 0.6646, lactating = 0.6837 p = 0.108]. Treating the dog in Kelusa vaccinated ~ 2 weeks before her puppies (as per Note 2(iii)) as lactating at vaccination, apart from time the model with the lowest AIC included upper outliers and retained lactation at vaccination only [observations = 437, intercept = 0.6617, lactating = 0.6959 p = 0.083].

Note 6: One additional linear model, fitted to two time points (day 180 and 360) with the intercept adjusted to day 30, with the lowest AIC retained covariates other than time. The model included natural log of the titre as the response variable and time, age, gender, pregnancy, lactation, body condition, clinical signs and generalised dermatitis as covariates (the same inclusion/exclusion criteria for lactation and generalised dermatitis as Models 1 and 2, see Notes 1, 2 and 4 above). The results were similar to Model 2 (i.e. the larger data set). With upper outliers and treating the dog in Kelusa vaccinated ~ 2 weeks before her puppies (as per Note 2(iii)) as not lactating at the time of vaccination, apart from time the model with the lowest AIC retained generalised dermatitis at the time of vaccination only [observations = 402, intercept = 0.8688, with generalised dermatitis = -0.3992 p = 0.012]; and, treating the dog in Kelusa vaccinated ~ 2 weeks before her puppies (as per Note 2(iii)) as lactating at vaccination, apart from time the model with the lowest AIC retained lactation and generalised dermatitis at vaccination [observations = 402, intercept = 0.8325, lactating = 0.6339 p = 0.137, with generalised dermatitis = -0.3705 p = 0.020]. Without upper outliers, apart from time the model with the lowest AIC retained generalised dermatitis at vaccination only [observations = 373, intercept = 0.5935, with generalised dermatitis = -0.3635 p= 0.014].
