# Supplementary material for: Achieving Population-Level Immunity to Rabies in Free-Roaming Dogs in Africa and Asia
Source: PLoS Negl Trop Dis. 2014 Nov 13;8(11):e3160. doi: 10.1371/journal.pntd.0003160 (PMC4230884; doi:10.1371/journal.pntd.0003160)
Supplement: Table S24 — Contingency tables for the covariates in the models detailed under Statistical methods in the Materials and Methods (see Tables S20, S21, S22, S23). (DOCX) [file pntd.0003160.s025.docx]

Table S24 Contingency tables of the covariates in the models detailed under *Statistical methods* in the Methods and materials (see Tables S20-S23); the tables show the maximum number of dogs with that factor level in the models that included upper outliers
