# Supplementary material for: Achieving Population-Level Immunity to Rabies in Free-Roaming Dogs in Africa and Asia
Source: PLoS Negl Trop Dis. 2014 Nov 13;8(11):e3160. doi: 10.1371/journal.pntd.0003160 (PMC4230884; doi:10.1371/journal.pntd.0003160)
Supplement: Table S25 — Dogs vaccinated by the Department of Livestock in Kelusa with Rabivet Supra 92. (DOCX) [file pntd.0003160.s026.docx]

Table S25 Dogs vaccinated by the Department of Livestock in Kelusa with Rabivet Supra 92

* ≥33rd month of life in December 2009; ᶧmonth of life in this range

Note: this table excludes two mature adults (one male and one female) vaccinated by the Department of Livestock in December 2009 that died prior to the commencement of blood sampling in June 2010
