# Supplementary material for: Achieving Population-Level Immunity to Rabies in Free-Roaming Dogs in Africa and Asia
Source: PLoS Negl Trop Dis. 2014 Nov 13;8(11):e3160. doi: 10.1371/journal.pntd.0003160 (PMC4230884; doi:10.1371/journal.pntd.0003160)
Supplement: Table S26 — The number of dogs in Zenzele with intestinal parasites on day 0. (DOCX) [file pntd.0003160.s027.docx]

Table S26 The number of dogs in Zenzele with intestinal parasites on day 0 (see Stext3)

| parasite | number of dogs diagnosed with the parasite |
| --- | --- |
|  |  |
|  |  |
| Ancylostoma | 103 |
|  |  |
| Coccidia | 2 |
|  |  |
| Spirocerca | 6 |
|  |  |
| Toxocara | 5 |
|  |  |
| Hymenolepis nana | 1 |
|  |  |

Note: only 3 (of the 107 dogs tested) were negative for parasites
