# Supplementary material for: Achieving Population-Level Immunity to Rabies in Free-Roaming Dogs in Africa and Asia
Source: PLoS Negl Trop Dis. 2014 Nov 13;8(11):e3160. doi: 10.1371/journal.pntd.0003160 (PMC4230884; doi:10.1371/journal.pntd.0003160)
Supplement: Text S1 — Sample selection and sampling technique for deep skin scrapes. (DOCX) [file pntd.0003160.s028.docx]

Stext1: Sample selection and sampling technique for deep skin scrapes

Deep skin scrapes (DSS) were taken on day 180 in Bali to investigate generalised demodicosis. Having to catch affected dogs by net precluded true random selection, therefore a convenience sample of 15 dogs in Kelusa and 17 in Antiga were selected from the pool of 168 dogs diagnosed with generalised dermatitis during the preceding survey. To estimate the true prevalence of demodicosis in free-roaming dogs with dermatitis a sample size of approximately 90 dogs was required [[1](#_ENREF_1)] [assuming an expected prevalence of 12% [[2](#_ENREF_2),[3](#_ENREF_3)], precision of 5%, and sensitivity of 90% and specificity of 99% based on operator experience and type of test [[4](#_ENREF_4)]]. Practical constraints limited testing to approximately 30 dogs with generalised dermatitis as a pilot study, considered sufficient to gauge the involvement of *Demodex spp.*

Lesions were arbitrarily classified as generalised according to the general literature on demodicosis [[5](#_ENREF_5),[6](#_ENREF_6)]. Dogs with generalised dermatitis had 12 or more localised lesions, and/or contiguous lesions that may have included complete involvement of an entire body region (e.g. face, flank), and/or complete involvement of two or more feet.

Multiple deep skin scrapes, approximately 2x2cm, were taken from each affected dog according to standard protocols [[6](#_ENREF_6)]. The cellular debris was placed into plain, coded 3ml blood tube and examined within 12 hours of sampling using a standard light microscope (from 4-40x magnification). Prior to microscopy, the samples were soaked in 10% potassium hydroxide for approximately 20 minutes to dissolve the cellular debris.

References

1. AusVet (2013) <http://epitools.ausvet.com.au/content.php?page=PrevalenceSS&HTP=0.1&HSENS=0.95&HSPEC=1&Popsize=170&Conf=0.95&Precision=0.05>. Epi Tools.

2. Rodriguez-Vivas RI, Ortega-Pacheco A, Rosado-Aguilar JA, E BGM (2003) Factors affecting the prevalence of mange-mite infestations in stray dogs of Yucatan, Mexico. Veterinary Parasitology 115: 61-65.

3. Nayak DC, Tripathy SB, Dey PC, Ray SK, Mohanty DN, et al. (1997) Prevalence of canine demodicosis in Orissa, India. Veterinary Parasitology 73: 347-352.

4. Fondati A, De Lucia M, Furiani N, Monaco M, Ordeix L, et al. (2009) Prevalence of *Demodex canis* - positive healthy dogs at trichoscopic examination. Veterinary Dermatology 21: 146-151.

5. Mueller RS (2004) Treatment protocols for demodicosis: an evidence based review. Veterinary Dermatology 14: 75-89.

6. Miller WH, Griffin CE, Campbell KL (2013) Muller & Kirk's Small Animal Dermatology 7th Edition. St. Louis, United States: Elsevier.
