# Supplementary material for: Achieving Population-Level Immunity to Rabies in Free-Roaming Dogs in Africa and Asia
Source: PLoS Negl Trop Dis. 2014 Nov 13;8(11):e3160. doi: 10.1371/journal.pntd.0003160 (PMC4230884; doi:10.1371/journal.pntd.0003160)
Supplement: Text S2 — Description of the covariates in the models detailed under Statistical methods in the Materials and Methods (see Table S19). (DOCX) [file pntd.0003160.s029.docx]

Stext2: Description of the covariates in the models detailed under *Statistical methods* in the Methods and materials (see Table S19)

The covariates were evaluated by direct observation, by the primary researcher and the enumerators, and by owner questionnaire. The questionnaire was developed and tested in February 2008 through community focus groups, including modified participatory rural appraisal (PRA) techniques [[1-3](#_ENREF_1)], and subsequent pilot studies. In Bali the questionnaires were bilingual (English and Bahasa with the Bahasa back-translated). Several languages were spoken in Johannesburg, including English, therefore the questions were written in English and the accuracy of the various translations checked regularly with the multi-lingual enumerators throughout the study period. The respondent was the person/s in the household that the householders collectively identified as most knowledge about the dog, which was not necessarily the owner. Respondents under 16 years of age were always interviewed with an adult present. Data collection was standardised through detailed enumerator training at the start of the study period for each enumerator and repeated on the first day of each survey. Surveys were undertaken every 6-12 weeks from March 2008 until April 2011. Each survey included the direct observations, by the primary investigator and enumerators, and the owner questionnaires. Apart from the primary researcher, all enumerators were local residents. All clinical examinations were undertaken by a qualified veterinarian.

The age of the dogs was reported by owners and/or visually assessed by the enumerators, including from the dentition in puppies and juveniles (i.e. dogs ≤ their 12th month of life [≤ ~52 weeks of age]) [[4](#_ENREF_4)] in Zenzele. The exact age (month of life) at vaccination was known for most dogs ≤ their 35th month in Zenzele, 33rd month in Kelusa, and 32nd month in Antiga (i.e. for dogs acquired from March 2008 as puppies or juveniles). Body condition includes the minimum and maximum score recorded during the surveys immediately prior to and following vaccination (i.e. within 6 weeks of vaccination). Body condition was evaluated using a standard 9-point (emaciated score 1 to obsess score 9) scoring system validated in adults [[5](#_ENREF_5)] and modified to assess body condition score (BCS) without palpation. The modified system was validated using dual energy x-ray absorptiometry in 71 dogs, including a small number of growing dogs [[6](#_ENREF_6),[7](#_ENREF_7)]. Each dog was body condition scored independently by two enumerators during each survey. The minimum and maximum scores were generally the same or one score point different (e.g. 3 and 3, or 3 and 4 respectively). Clinical signs were observed by the primary researcher and enumerators at the time of vaccination or during the survey immediately prior to vaccination. As part of the owner questionnaire, owners reported clinical signs they had observed during the previous 7 days and 3 months based on a set of pictures, each of a dog with a different clinical sign. With the exception of generalised dermatitis, clinical signs were those associated with serious illness and likely to cause weight loss, e.g. vomiting, lethargy. Almost all of the dogs with generalised dermatitis were in Bali, and were the majority of those with observed clinical signs. There was insufficient variation in reported protein intake in Zenzele for analysis. In Bali, protein intake (either never/rarely=0 or more frequent than never/rarely=1) reported during the survey immediately prior to vaccination was generally consistent with that fed throughout the study period.

References

1. Chambers R (1994a) The origins and practice of participatory rural appraisal. World Development 22: 953-969.

2. Kumar S (2007) Methods for community participation. A complete guide for practitioners. Warwickshire, United Kingdom: Intermediate Technology Publications Ltd.

3. Chambers R (2007) From PRA to PLA and pluralism: practice and theory. United Kingdom: Institute of Development Studies.

4. Dyce KM, Sack WO, Wensing CJG (1987) Textbook of Veterinary Anatomy; Pedersen D, editor. Philadelphia, U.S.A: Saunders.

5. Laflamme D (1997) Development and validation of a body condition score system for dogs. Canine Practice 22: 10-15.

6. German AJ, Holden SL, Moxham GL, Holmes KL, Hackett RM, et al. (2006) A simple, reliable tool for owners to assess the body condition of their dog or cat. The Journal of Nutrition 136: 2031S-2033S.

7. German AJ, Holden SL. Subjective estimation of body condition can predict body fat mass as well as condition scoring with an established 9-point scale; 2006; Birmingham, United Kingdom. pp. 508.
