# Supplementary material for: Achieving Population-Level Immunity to Rabies in Free-Roaming Dogs in Africa and Asia
Source: PLoS Negl Trop Dis. 2014 Nov 13;8(11):e3160. doi: 10.1371/journal.pntd.0003160 (PMC4230884; doi:10.1371/journal.pntd.0003160)
Supplement: Text S3 — Sample selection for faecal analysis (see Table S26). (DOCX) [file pntd.0003160.s030.docx]

Stext3: Sample selection for faecal analysis (see Table S26)

A prevalence of ~90% was expected for intestinal parasites in Zenzele, particularly *Anycylostoma spp.* [[1](#_ENREF_1),[2](#_ENREF_2)]. In order to estimate true prevalence with a precision of 5% and test sensitivity and specificity of at least 95%, a sample of approximately 120 dogs was required. This estimate happened to coincide with the number of dogs (n=107) randomly selected to assess intestinal parasitism as a pilot study.

One dog was positive for *Hymenolepis nana*, a tapeworm found in humans and rodents, which was probably acquired from eating a rodent [[3](#_ENREF_3)].

References

1. Minnaar WN, Krecke RC (2001) Helminths in dogs belonging to people in a resource-limited urban community in Gauteng, South Africa. Onderstepoort Journal of Veterinary Research 68: 111-117.

2. Minnaar WN, Krecke RC, Rajput JI (1999) Helminth parasites of dogs from two resource-limited communities in South Africa. Journal of the South African Veterinary Association 70: 92-94.

3. Palmer SR, Soulsby EJL, Torgerson PR, Brown DWG (2011) Oxford textbook of zoonoses: biology, clinical practice and public health control, 2nd edition. United Kingdom: Oxford University Press.
